# An Approach to Potentially Increasing Adoption of an Artificial Intelligence–Enabled Electronic Medical Record Encounter in Canadian Primary Care: Protocol for a User-Centered Design

**DOI:** 10.2196/54365

**Published:** 2024-07-18

**Authors:** Krizia Mae Francisco, Catherine M Burns

**Affiliations:** 1 Department of Systems Design Engineering University of Waterloo Waterloo, ON Canada

**Keywords:** primary care, electronic medical record, EMR, artificial intelligence, AI, contextual design, user-centered, design, electronic health record, EHR, Canada, Canadian, primary care, physicians, burnout, user, users, tools, provider-centered design, decision-making, AI tools, technology acceptance model, TAM

## Abstract

**Background:**

Primary care physicians are at the forefront of the clinical process that can lead to diagnosis, referral, and treatment. With electronic medical records (EMRs) being introduced and, over time, gaining acceptance by primary care users, they have now become a standard part of care. EMRs have the potential to be further optimized with the introduction of artificial intelligence (AI). There has yet to be a widespread exploration of the use of AI in primary health care and how clinicians envision AI use to encourage further uptake.

**Objective:**

The primary objective of this research is to understand if the user-centered design approach, rooted in contextual design, can lead to an increased likelihood of adoption of an AI-enabled encounter module embedded in a primary care EMR. In this study, we use human factor models and the technology acceptance model to understand the results.

**Methods:**

To accomplish this, a partnership has been established with an industry partner, TELUS Health, to use their EMR, the collaborative health record. The overall intention is to understand how to improve the user experience by using user-centered design to inform how AI should be embedded in an EMR encounter. Given this intention, a user-centered approach will be used to accomplish it. The approach of user-centered design requires qualitative interviewing to gain a clear understanding of users’ approaches, intentions, and other key insights to inform the design process. A total of 5 phases have been designed for this study.

**Results:**

As of March 2024, a total of 14 primary care clinician participants have been recruited and interviewed. First-cycle coding of all qualitative data results is being conducted to inform redesign considerations.

**Conclusions:**

Some limitations need to be acknowledged related to the approach of this study. There is a lack of market maturity of AI-enabled EMR encounters in primary care, requiring research to take place through scenario-based interviews. However, this participant group will still help inform design considerations for this tool. This study is targeted for completion in the late fall of 2024.

**International Registered Report Identifier (IRRID):**

DERR1-10.2196/54365

## Introduction

### Background

In the Canadian health care system, primary care clinicians have played an integral role in providing continuity of patient care. Primary care clinicians are the first point of contact for patients and the health care system; their scope of work includes illness prevention, health promotion, diagnosis, treatment, rehabilitation, and counseling [1]. Approximately 93% of Canadian primary care physicians are using electronic medical records (EMRs) in their practice [2], which is a steep increase from the 37% of EMR use in 2009 [3]. An EMR can be defined as “a secure software system and its associated database. It is the record that primary care clinicians use within their practice environment to capture patient information such as a patient’s family history, lab requests and results, cancer screening tests, emergency room visits, prescriptions, and more” [4]. EMRs were introduced and, over time, have gained acceptance and credibility for improving a primary care clinician's ability to provide care [5-7]. They have now become a standard part of care and are further supported through professional colleges and regulatory policies. EMRs have the potential to be further optimized with the introduction of artificial intelligence (AI). It is important to highlight how we can optimize and design AI in the primary care setting to ensure that it is ultimately supporting primary care delivery [5].

AI arguably has endless possibilities for application, which is what makes it both incredibly exciting and difficult to implement. With varying expectations and levels of comprehension, it is important to identify various stakeholders in the health care system and understand their expectations and intentions regarding AI in health care. The generalist approach and lack of specialization in a single system of the body make primary care a more difficult sector to embed AI into, in contrast to specific specialties such as oncology or diagnostic imaging. These areas of specialty allow data to be more specific, which makes the exploration of the use of AI more narrowly scoped than if it were primary health care [8]. The data available in health care are considered sensitive and highly personal. The way AI presents information to a clinician in an interaction has the potential to impact behaviors, including how decision-making is made [9-11]. Understanding human acceptance and the potential impact of a clinical encounter with the introduction of AI is a key milestone in enabling the industry to effectively predict the potential successes and barriers to AI-enabled products and services. A scoping review captured the various research foci related to AI in primary care research [8]. Its findings indicate that there has yet to be a widespread exploration of the use of AI in primary health care and how clinicians envision AI use to encourage further uptake [8]. This study supports the notion that research for primary care and AI is at an early stage of maturity [8]. Given the state of maturity, identifying the factors that influence the adoption of AI-enabled features in an EMR is essential to ensuring its acceptability by primary care clinicians.

The contextual design process will be used as the foundation of this research. Contextual design was first invented in 1988 and is continuously used in a wide variety of industries to drive innovative design [12]. Contextual design is a user-centered design process that uses techniques to analyze user data, drive ideation from the data, develop a design based on the data, and iterate these designs with the end users [12].

There are 3 distinct phases to contextual design as follows:

- Phase 1: Gathering user data. User-centered design recognizes that innovation starts with an understanding of the user by gaining an in-depth understanding of their tasks, motivations, intents, strategies, and detailed steps [12].
- Phase 2: Deriving insight from the data. Qualitative methods of understanding the data collected in Phase 1 are used to drive inductive insight into the design. This phase is further supported by other contextual design tools, such as affinity diagrams or experience models, to better articulate user design requirements [12].
- Phase 3: Taking data to design. This key phase involves translating the data to drive design thinking and visioning. Prototypes and mock-ups are redesigned with users to better fit their activities [12].

This process will be supported by the technology acceptance model (TAM) as a frame of reference. Although the TAM model has evolved in the last 30 years, the core of TAM remains focused on perceived ease of use and perceived usefulness [13]. Perceived ease of use and perceived usefulness influence attitudes and intentions, which can influence and predict user acceptance and adoption [13].

There is great potential and significant risk to advancing AI technologies in health care. The risk of embedding AI in an EMR encounter is disrupting an actively accepted and used tool that they perceive as a benefit to their performance [6,7]. Inappropriately introducing AI in the clinical encounter could impact its perceived usefulness, which could further deter the willingness and uptake of AI-enabled tools by primary care clinicians [14,15].

For the context of this research, the users are primary care health care providers (eg, primary care physicians, and nurse practitioners) in Ontario, Canada.

The primary objective of this research is to understand if the user-centered design approach, rooted in contextual design, can lead to an increased likelihood of adoption of an AI-enabled encounter module embedded in a primary care EMR. We are using human factor models and TAM to understand the results.

### TELUS Health Partnership

To accomplish this, a partnership has been established with an industry partner, TELUS Health, to use their EMR, the collaborative health record (CHR). The CHR is TELUS Health’s cloud-based EMR solution. The CHR has standard features such as the cumulative patient profile, free-text charting, custom forms, and electronic booking. However, it does have advanced functions, including streamlined clinical encounters, embedded virtual care, and real-time clinical and business intelligence.

The applicability of this proposed research is vast. This proposed work can demonstrate how to better optimize the use of AI-enabled products and services in the primary care setting, which is important given how the health care environment continues to evolve today. This can act as a key study that can inform some fundamental approaches to incorporating AI-enabled tools or services.

## Methods

### Overview

The approach of user-centered design requires qualitative interviewing to gain a clear understanding of users’ approaches, intentions, and other key insights to inform the design process. This approach will be supported by qualitative methods to achieve the research objective. Several similar studies use a qualitative approach for user-centered design to inform technology acceptance in health care [14,16-19].

Preread material on AI and its capabilities within Canadian primary care has been developed for primary care clinicians to review in advance of the interviews.

### Phase 1: Initial Interviews

All interviews will take place individually using a semistructured format comprising a standard set of scenarios with the ability to ask follow-up questions based on the participant's answer. This approach to interviewing enables a balance to provide purpose to the interview through the standard question set while also focusing the conversation on ideas and responses that can significantly inform the research and design considerations.

Several qualitative studies are conducted to inform contextual design in a clinical interaction. A number of these studies have demonstrated that anything over a total of 20 participants did not produce any significantly unique answers, which will inform our ability to reach data saturation with similar methods [16-18,20]. For this reason, we will recruit up to 20 participants in total. The participants will be comprised of primary care clinicians currently practicing in Ontario, Canada. Each participant will be asked to participate in the interview structure outlined below, with the potential for subsequent interviews to be requested based on input gathered at the secondary interview, primarily focused on validating findings from the first interview. All interviews will occur virtually using Microsoft Teams, where audio will be recorded and automatically transcribed through Microsoft Teams. Immediately after the interview, the interviewer will correct minor errors in the transcription.

### Scenarios With the Primary Care Encounter Module

**Figure 1:**
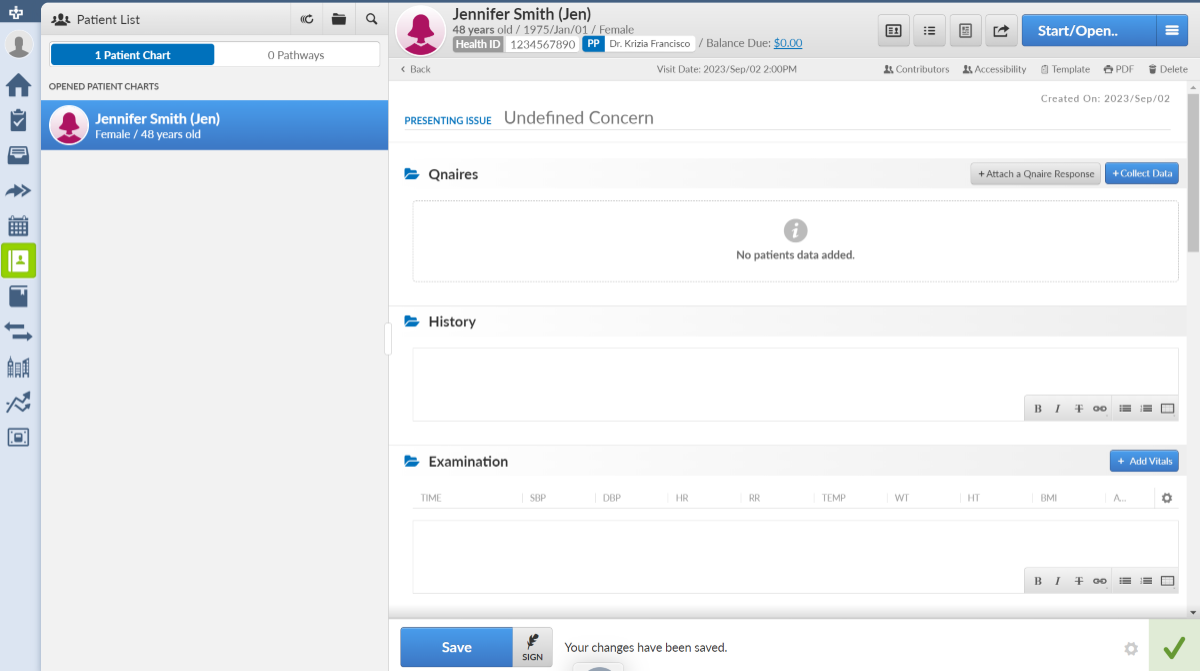
The encounter module in the TELUS CHR in an empty state. CHR: collaborative health record.

The goal is to understand a clinician’s decision-making process through a clinical interaction with a patient and what key elements of workflow exist (eg, when do they document the clinical encounter, what do they consider distracting on their EMR screen, etc). Given the breadth and ambiguity of presentation in primary care, specific scenarios have been chosen focused on general approaches to primary care physician workflows. The primary module within the CHR that will be used for these interviews is the “Encounter.” The Encounter module is where primary care clinicians spend the majority of their time documenting, even during a clinical visit. It captures information on patient responses to questionnaires, health history, examinations, assessment and planning, prescriptions, attachments, referrals, injections, follow-up items, and billing. Encounters require comprehensive information capture, making it an ideal module to focus on for added AI and its potential to impact how a physician captures a clinical encounter. Figure 1 shows the current structure of the Encounter module within the CHR.

### Primary Care Physician Initial Interviews (Duration: 60 Minutes Total)

#### Part 1 (30 Minutes) Current State: Clinician

##### Overview

There will be an initial interview with the primary care physicians to understand their current approach to documenting clinical encounters during a visit. The goal is to establish a workflow of what clinicians use in their existing EMR for that particular scenario.

##### Scenario 1

Taylor Jones, a female patient aged 29, called your office 2 weeks ago requesting a visit related to her mental health. In preparation for her appointment, she was given a GAD7 to fill out at home; the results were then sent to your office, with a score of >15, indicating severe anxiety. This patient is now sitting in your office, and the encounter screen is open on your screen. What flow would you engage in? (eg, which sections would you expand first, what would you chart, and what are the questions you ask next).

##### Scenario 2

Alice Smith, a female patient aged 48, called your office with a suspected UTI. The last time she was prescribed an antibiotic for a UTI was over 2 years ago. The patient is now sitting in your office with this encounter screen open on your screen. What flow would you engage in? (eg, which sections would you expand first, what would you chart, and what are the questions you ask next).

#### Part 2 (30 Minutes; Scenario Exposure CHR Enabled With AI)

##### Overview

**Figure 2:**
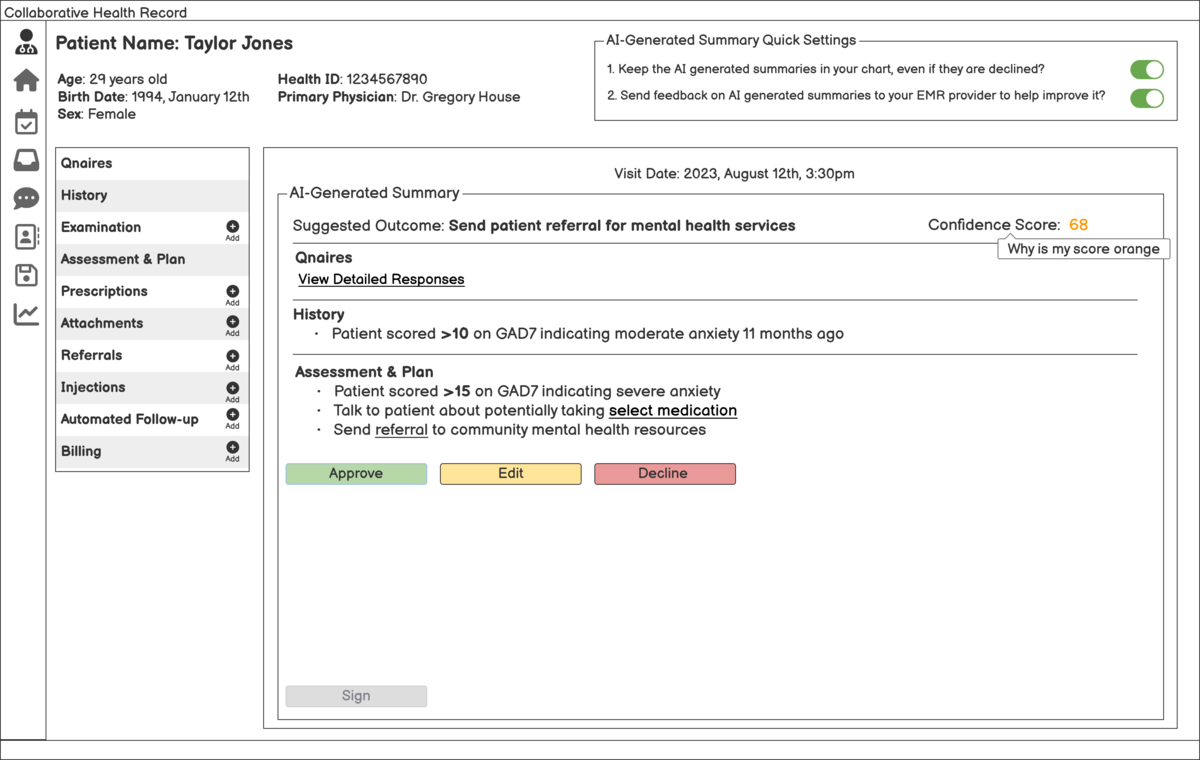
The AI-enabled version of the encounter in the CHR shown to clinicians during part 2 of the first interview. AI: artificial intelligence; CHR: collaborative health record; GAD7: Generalized Anxiety Disorder-7 Scale.

Physicians will be presented with the same scenarios as in the first interview, but now with an AI-enabled version of the CHR encounter. The objective is to understand how physicians will interact with the AI and whether it prompts differences in their workflow. Figure 2 shows example mock-ups that will be shown to primary care clinicians, demonstrating an AI-enabled version of the encounter module in the CHR. Scenarios 3 and 4 are the same as scenarios 1 and 2, except for adding an AI-enabled EMR encounter.

##### Scenario 3

Taylor Jones, a female patient aged 29, called your office 2 weeks ago requesting a visit related to her mental health. In preparation for her appointment, she was given a GAD7 to fill out at home; the results were then sent to your office, with a score of >15, indicating severe anxiety. Your EMR is now supported by AI, and before the patient’s appointment, it processes the results and prepopulates your encounter. This patient is now sitting in your office, and the encounter screen is open on your screen. What flow would you engage in with the following screen? (eg, which sections would you expand first, what would you chart, and what are the questions you ask next).

##### Scenario 4

Alice Smith, a female patient aged 48, called your office with a suspected UTI. The last time she was prescribed an antibiotic for a UTI was over 2 years ago. Your EMR is now supported by AI, and before the patient’s appointment, it processes the results and prepopulates your encounter. The patient is now sitting in your office with this encounter screen open on your screen. What flow would you engage in with the following screen? (eg, which sections would you expand first, what would you chart, and what are the questions you ask next).

This mock-up introduces the concept of an AI-generated summary for primary care clinicians. Key differences between this summary and a traditional EMR encounter are provided in Textbox 1.

**Key differences between the AI-generated summary and a traditional electronic medical record (EMR) encounter.**

- A numeric confidence score. It is intentionally unspecified whether or not the suggested outcome confidence score is based on a large language model data set informed by best practice, the clinician’s practice, or the organization’s data set, as this is also a key design consideration that can be further explored in the interview.
- This also introduces the concept of “approve, edit, or decline” buttons in the EMR encounter to further understand how primary care clinicians want to interact with AI from a liability lens (eg, if a clinician declines what the AI is suggesting, do they want it to remain a part of the record? Or, if a clinician decides to edit the summary, do they want the chart to delineate what was AI-generated versus what was manually edited? Or do they view the note after it’s been approved as their record is not distinguishable from what the AI suggested?).
- There are underlined actionable steps in this encounter (eg, “select medication”), demonstrating that the AI has not taken the further step of assigning a more specific medication to prescribe. Further probing clinicians on whether or not they think the AI has taken its suggestion too far, or not far enough.

### Phase 2: Analysis and Reporting

The data collected throughout this research will be qualitatively analyzed concurrently with the interviews. This process aims to be deductive with the approach, supported by TAM, codes being sorted into one of 3 categories: perceived usefulness, perceived ease of use, and general attitudes towards using AI in health care practice. The coding techniques being applied are a result of the goals of the research being conducted. The codes discovered throughout the research will inform the various redesign approaches to an AI-enabled version of the CHR encounter. To provide further guidance related to AI-enabled primary care EMRs, key principles from design can be informed by a thematic analysis that results from the codes within the determined categories. A reporting guideline will be completed, the consolidated criteria for reporting qualitative research (COREQ), to further support the qualitative analysis [21].

The analysis will take place in the following way (Textbox 2):

The second and third steps in Textbox 2 are iterative and will be visited and then revisited numerous times until they become more refined and, as a result, more conceptual.

**Qualitative analysis.**

- **Preliminary coding:** at this stage, the coding does not have to be accurate or final. The goal is to familiarize myself with the data that have been collected. Immediately after an interview, I will conduct preliminary coding, highlighting any significant quotes. At this stage, capturing ideas for analytic consideration while the study progresses is essential. These are vital first impressions that can provide a transitional link between raw data and codes [22].
- **First cycle coding:** the goal of the first cycle, once all interviews have been conducted, is to provide an initial analysis. Semantic coding will be used to do this. Specific segments of the interview data will be sectioned and labeled using either a word or a short phrase from that particular section of the data. Semantic codes do not require any interpretation beyond what the participants have said. These codes can be viewed as a description that only represents the content of the data as it has been communicated by the participant [22]. It is important at this stage that these initial codes provide enough context.
- **Sorting into categories:** categories were established based on the TAM and are indicative of the consolidated meaning that is being analyzed in the data. I am hopeful that the categories developed will be informative about the various design changes that can occur with an AI-enabled version of the CHR encounter.
- **Thematic analysis:** the outcome of the second and third steps is theme development. Coding and theming are not mutually exclusive procedures [22]. Themes are more descriptive versions of the categories discovered in the data. Theming data categorically provides descriptive detail about the patterns observed and constructed [22]. With the thematic analysis becoming a macro-representation of the patterns, these can be translated into fundamental principles that inform the future design of other AI-enabled digital tools in the primary care setting.

### Phase 3: Develop User-Centered Design Requirements

The contextual inquiry methodology will be used to develop the user-centered design requirements. The principles of contextual inquiry enable the right type of interviewing to elicit design requirements, which include understanding the context, developing a partnership with the users, and engaging in the interpretation process. This method requires the researcher to assume a role similar to an “apprentice,” where those being interviewed are experts in their own experiences. The introduction of AI technology within the CHR encounter for these common primary care visits is just 1 key component of a much broader process and experience that needs to be understood and considered. Contextual design is driven by the fact that products are always part of a larger picture, making this a suitable method to complement the proposed research [12]. The researcher minimizes the introduction of bias by moving through a continuous interpretation process; by engaging users in the interview process in sharing interpretation, they are typically quick to correct any misguided or misinterpretations that are shared.

The interview will provide initial exposure to an AI-enabled version of the CHR. Participants will be prompted to provide feedback on what they do and do not like about the interface or what they do and do not find helpful as a part of the interface. The interviews will also highlight critical experiences from participants and, through the contextual design approach, reveal intentions of what is not explicitly being said. This information will be interpreted and translated into design requirements which the redesigned mock-up interfaces will be based on. This information will be used to build personas to inform how these designs will be mocked up.

### Phase 4: Redesign of an AI-Enabled Encounter Module in the CHR

Both categories and themes identified in the thematic analysis will be used to inform the redesign of an AI-enabled encounter module within the CHR. The goal is for the data to inform user-centered design requirements that accurately reflect the motivations, thoughts, and attitudes of the particular audience or participant. For example, what features on the output may be seen as adding friction and not providing value or purpose that can be presented differently?

Redesigns of the AI-enabled CHR will be completed in a low-fidelity wireframe using Balsamiq, focusing on functional design rather than aesthetic design. After phase 4 is completed, high-resolution wireframes that are consistent with TELUS Health’s branding will be completed. TELUS Health will be involved in the iterative design process and will review the mock-up iterations before engaging in a usability study to refine designs further.

### Usability Study

A usability study is required at this point in the process to select which of the redesigned AI-enabled mock-up interfaces are most usable and accepted. Based on other usability studies conducted with similar methods, 8-12 participants are considered reliable [23,24].

Participants will be given access to interactive wireframes using Balsamiq (Balsamiq Studios), accompanied by a system usability scale. As participants navigate the wireframes, open-ended questions will be asked to encourage further feedback on the redesigned interfaces. This system usability scale will be used to determine which of the redesigned outputs are best. Once this has been completed, the best designs will be mocked up using Figma (Figma Inc), a higher fidelity software that can more consistently reflect TELUS Health’s CHR initial user-interface branding. These will then be presented in the secondary interview process. This method is well supported, efficient, and has a considerable amount of existing research to support its reliability.

### Phase 5: Validate in a Secondary Interview (Duration: 60 Minutes)

Data gathered from the initial interview will be used to inform potential improvements that can be made. Alternative AI-enabled EMR encounter screens will be redesigned, mocked up, and presented to clinicians along with the scenarios they were presented during the initial interview. The goal is to understand whether or not the redesigned output was seen as an improvement from the initial designs in the first interview.

### Recruitment of Participants

Targeted communications with well-known primary care networks will be used for recruitment (eg, primary care digital health networks, Association for Family Health Teams), in addition to direct emails to physicians or direct contact through social media platforms such as LinkedIn. The inclusion criteria for this study related to physicians is that they are primary care physicians actively practicing in Ontario. In addition, any other requirements outlined by the University of Waterloo's Office of Research Ethics will be implemented upon review of this research protocol application.

All participating primary care clinicians will be provided a one-time CAD $75 honorarium in the form of a VISA gift card in exchange for their valuable time and input.

### Ethical Considerations

This study has been reviewed and received ethics clearance through the University of Waterloo Research Ethics Board (43922). This application includes the formal consent letter that will be used to support the study and reiterate the confidentiality of participation, in addition to an oral consent script to be used with participants. Participants will be informed that transcripts of their interviews will be recorded and stored on an encrypted hard drive for a 7-year retention period in alignment with the University of Waterloo's best practice guidelines. Based on the feedback provided by this office, there may be minor changes made to the approach of this research study.

## Results

As of March 2024, a total of 14 primary care clinicians have participated in this protocol, and their data have undergone the first phase of coding. The research team is assessing data saturation and strengthening the underlying theories that support this work related to the TAM. The results will be made available in a follow-up publication.

## Discussion

### Principal Findings

Some limitations need to be acknowledged related to the approach of this study. There is a lack of market maturity for AI-enabled EMRs in primary care, requiring research to take place through scenario-based interviews. Due to the limitation of risky investment in the actual development of an AI-enabled CHR, the proposed research would take place through scenario-based interviews with primary care clinicians and controlled exposure to mock-up interfaces of an AI-enabled CHR. This method can present several limitations. Given the lack of maturity in the use of AI-enabled EMRs in the Canadian primary care setting, it is difficult to provide users with a novel tool and not provide them with the guidance required to understand its use in a targeted context. This proposed research can continue to inform how standards compliance should be designed for AI-enabled tools, which can build credibility with clinicians over time.

There is also the limitation that scenario-based interviews limit our ability to observe and understand a person's natural use of the tool. There is still an opportunity to observe critical components of these tools that may cause a behavior change that can be isolated from other features of the proposed tool (eg, how only the output of a tool impacts a person's ability to make a decision). As the development of AI-enabled tools in primary care continues to mature, so will the ability to study and redesign their broader impact on clinical interaction.

In addition, the types of clinicians who choose to volunteer for this study may be more technically literate, which may not be representative of the general population’s abilities or feedback related to using an AI-enabled tool in a diagnostic context.

### Conclusions

This participant group will still help inform design considerations for this tool. However, if AI-enabled tools in the primary care domain gain more credibility, work needs to be done to understand the design implications for less tech-literate users. The proposed scenarios and methods will still gather the information required to inform key research questions and future design principles for AI-enabled tools in the primary care setting.

Research for primary care and the use of AI is at an early stage of maturity [8]. This research can contribute to assessing the real-world implications on primary care. This study is targeted for completion in late fall of 2024.

## Abbreviations

AI: artificial intelligence
CHR: collaborative health record
COREQ: consolidated criteria for reporting qualitative research
EMR: electronic medical record
TAM: technology acceptance model

## Data Availability

The data sets generated and analyzed during this study are not publicly available due to the consent agreement that was established with all participants.

## References

[1] (2024). Primary Care Payment Models in Ontario.

[2] Johnson T International survey results reveal challenges experienced by family doctors. CIHI.

[3] Canadian Institute for Health Information (2020). How Canada Compares: Results From the Commonwealth Funds 2019 International Health Policy Survey of Primary Care Physicians.

[4] Ontario MD (2024). What is an EMR?. EMR Certification Program FAQ.

[5] Ayanso A, Herath TC, O'Brien N (2015). Understanding continuance intentions of physicians with electronic medical records (EMR): an expectancy-confirmation perspective. Decis Support Syst.

[6] Jones M, Koziel C, Larsen D, Berry P, Kubatka-Willms E (2017). Progress in the enhanced use of electronic medical records: data from the Ontario experience. JMIR Med Inform.

[7] Raymond L, Paré G, Ortiz de Guinea A, Poba-Nzaou P, Trudel M, Marsan J, Micheneau T (2015). Improving performance in medical practices through the extended use of electronic medical record systems: a survey of Canadian family physicians. BMC Med Inform Decis Mak.

[8] Kueper JK, Terry AL, Zwarenstein M, Lizotte DJ (2020). Artificial intelligence and primary care research: a scoping review. Ann Fam Med.

[9] Baron R, Perrot S, Guillemin I, Alegre C, Dias-Barbosa C, Choy E, Gilet H, Cruccu G, Desmeules J, Margaux J, Richards S, Serra E, Spaeth M, Arnould B (2014). Improving the primary care physicians' decision making for fibromyalgia in clinical practice: development and validation of the fibromyalgia detection (fibrodetect®) screening tool. Health Qual Life Outcomes.

[10] Henselmans I, Van Laarhoven HW, Van der Vloodt J, De Haes HC, Smets EM (2017). Shared decision making about palliative chemotherapy: a qualitative observation of talk about patients' preferences. Palliat Med.

[11] Tsai C, Liu C, Lin H, Lin T, Kuo K, Lin J, Chen C, Lee M (2023). Implementation of a patient-centered mobile shared decision making platform and healthcare workers' evaluation: a case in a medical center. Inform Health Soc Care.

[12] Holtzblatt K, Beyer H (2017). Contextual Design: Design for Life.

[13] Davis F, Granic A (2024). The Technology Acceptance Model: 30 Years of TAM.

[14] Holden RJ, Karsh B (2010). The technology acceptance model: its past and its future in health care. J Biomed Inform.

[15] Davis FD (1993). User acceptance of information technology: system characteristics, user perceptions and behavioral impacts. Int J Man Mach Stud.

[16] Zhu M, Zhang Y (2022). Medical and public health instructors' perceptions of online teaching: a qualitative study using the technology acceptance model 2. Educ Inf Technol (Dordr).

[17] Vereenooghe L, Trussat F, Klose K (2020). Applying the technology acceptance model to digital mental health interventions: a qualitative exploration with adults with intellectual disabilities. J Ment Health Res Intellect Disabil.

[18] Abdullah A, Liew SM, Hanafi NS, Ng CJ, Lai PSM, Chia YC, Loo CK (2016). What influences patients' acceptance of a blood pressure telemonitoring service in primary care? A qualitative study. Patient Prefer Adherence.

[19] Yarbrough AK, Smith TB (2007). Technology acceptance among physicians: a new take on TAM. Med Care Res Rev.

[20] McAlearney AS, Schweikhart SB, Medow MA (2004). Doctors' experience with handheld computers in clinical practice: qualitative study. Br Med J.

[21] Tong A, Sainsbury P, Craig J (2007). Consolidated criteria for reporting qualitative research (COREQ): a 32-item checklist for interviews and focus groups. Int J Qual Health Care.

[22] Saldan J (2021). The Coding Manual for Qualitative Researchers.

[23] Alroobaea R, Mayhew P (2014). How many participants are really enough for usability studies?.

[24] Macfield R (2021). How to specify the participant group size for usability studies: a practitioners guide - jux. JUX - The Journal of User Experience.

